# A Wearable Vibratory Device (The Emma Watch) to Address Action Tremor in Parkinson Disease: Pilot Feasibility Study

**DOI:** 10.2196/40433

**Published:** 2023-10-23

**Authors:** Alissa Pacheco, Tempest A van Schaik, Nadzeya Paleyes, Miguel Blacutt, Julio Vega, Abigail R Schreier, Haiyan Zhang, Chelsea Macpherson, Radhika Desai, Gavin Jancke, Lori Quinn

**Affiliations:** 1 Teachers College Columbia University New York, NY United States; 2 Microsoft Research Cambridge United Kingdom; 3 Department of Medicine University of Pittsburgh Pittsburgh, PA United States

**Keywords:** Parkinson’s disease, action tremor, Emma Watch, vibration, haptic feedback, handwriting, drawing, spirals, hand function

## Abstract

**Background:**

Parkinson disease (PD) is a neurodegenerative disease that has a wide range of motor symptoms, such as tremor. Tremors are involuntary movements that occur in rhythmic oscillations and are typically categorized into rest tremor or action tremor. Action tremor occurs during voluntary movements and is a debilitating symptom of PD. As noninvasive interventions are limited, there is an ever-increasing need for an effective intervention for individuals experiencing action tremors. The Microsoft Emma Watch, a wristband with 5 vibrating motors, is a noninvasive, nonpharmaceutical intervention for tremor attenuation.

**Objective:**

This pilot study investigated the use of the Emma Watch device to attenuate action tremor in people with PD.

**Methods:**

The sample included 9 people with PD who were assessed on handwriting and hand function tasks performed on a digitized tablet. Tasks included drawing horizontal or vertical lines, tracing a star, spiral, writing “elelelel” in cursive, and printing a standardized sentence. Each task was completed 3 times with the Emma Watch programmed at different vibration intensities, which were counterbalanced: high intensity, low intensity (sham), and no vibration. Digital analysis from the tablet captured kinematic, dynamic, and spatial attributes of drawing and writing samples to calculate mathematical indices that quantify upper limb motor function. APDM Opal sensors (APDM Wearable Technologies) placed on both wrists were used to calculate metrics of acceleration and jerk. A questionnaire was provided to each participant after using the Emma Watch to gain a better understanding of their perspectives of using the device. In addition, drawings were compared to determine whether there were any visual differences between intensities.

**Results:**

In total, 9 people with PD were tested: 4 males and 5 females with a mean age of 67 (SD 9.4) years. There were no differences between conditions in the outcomes of interest measured with the tablet (duration, mean velocity, number of peaks, pause time, and number of pauses). Visual differences were observed within a small subset of participants, some of whom reported perceived improvement. The majority of participants (8/9) reported the Emma Watch was comfortable, and no problems with the device were reported.

**Conclusions:**

There were visually depicted and subjectively reported improvements in handwriting for a small subset of individuals. This pilot study was limited by a small sample size, and this should be taken into consideration with the interpretation of the quantitative results. Combining vibratory devices, such as the Emma Watch, with task specific training, or personalizing the frequency to one’s individual tremor may be important steps to consider when evaluating the effect of vibratory devices on hand function or writing ability in future studies. While the Emma Watch may help attenuate action tremor, its efficacy in improving fine motor or handwriting skills as a stand-alone tool remains to be demonstrated.

## Introduction

Parkinson disease (PD) is a progressive neurodegenerative disease caused by the degeneration of dopamine in the substantia nigra and striatum areas of the brain that results in a decrease in the ability to control and coordinate movement [1,2]. PD currently affects more than 6 million people worldwide [3] and the incidence of PD is expected to double in the next 2 decades [2]. The cause of PD appears to be multifactorial, with behavioral, environmental, genetic, and lifestyle factors playing a role [2]. PD is characterized by both motor and nonmotor symptoms, however, the cardinal features include rigidity, tremor, bradykinesia, and postural instability [4]. While many forms of tremor may be present in PD, action tremor, which includes postural, isometric, and kinetic tremor [5,6], occurs during active, voluntary movement and is an impairment that affects writing, hand function, activities of daily living, and quality of life [4,7]. Furthermore, people with PD experience psychosocial implications resulting from their tremor, including negatively impacted relationships, self-image, and overall well-being [8]. Action tremor is reported in 46% of individuals with Hoehn and Yahr Stages 1 and 2, and up to 93% of people in Stages 1-5 [7].

The pathophysiology of action tremor is uncertain but has a clear difference from other motor symptoms of PD [9] as it is thought to be modulated by nondopaminergic pathways and does not correlate with dopamine depletion in the striatum; rather, serotonin, noradrenaline, and acetylcholine appear to play more of a role [9]. It may be caused by oscillations that occur within internal sensorimotor feedback circuits during movement [10,11] or by abnormal activity within the basal ganglia and cerebello-thalamo-cortical circuit [9,12] Action tremor frequency usually displays a 1.5 Hz higher frequency than that of rest tremor, which is ~4-6 Hz [9]. Interventions for the management of action tremor have been limited. Deep brain stimulation to the subthalamic nucleus and globus pallidus have shown improvement in action tremor severity at 6 and 12 months post implantation, but is invasive, can lead to adverse events, and is not suitable for everyone [11]. Dopaminergic medications also have limited efficacy in improving action tremor-related motor dysfunction [5,8,9,13], leaving a pressing need to address these functional sequelae.

Equivocal findings have been reported for total body vibration to improve motor function in people with PD [14], however recent studies suggest targeted vibration methods may be beneficial [15]. High-frequency vibration stimulation (also known as haptic feedback), along with medication, have improved movement initiation, movement speed, precision, and decreased tremor for people with PD [15-17]. Vibration is a form of sensory stimulation that results in increased sensory input and activation of the muscle spindle fibers, and may improve neuromotor functions in people with sensorimotor deficits [18]. Providing high frequency vibration over the forearm activates the muscle spindles and interrupts the central nervous system’s interpretation of the proprioceptive position of the limb in space, interpreting the vibration as sensory information and producing a muscle contraction [15].

Use of haptic feedback may reduce resting tremor and is considered safe and well tolerated when delivered in short durations via wearable devices, but its effects on action tremor has not been well studied [19,20]. The Emma Watch is a wrist-worn wearable device developed by Microsoft Research that provides constant high frequency vibration to each side of the wrist; preliminary findings suggest it may reduce movement speed and improve precision of performance in drawing and tracing tasks in people with PD [17]. However, efficacy of the Emma Watch on people with PD is unclear; therefore, this pilot study evaluated the use of the Emma Watch in people with PD who present with symptomatic and disruptive action tremor during handwriting, drawing, and hand function tasks.

## Methods

### Participants

In total, 9 people with PD were recruited for this pilot study, which began just prior to the COVID-19 pandemic. We had expected to enroll a total of 20 participants; however, human subjects research was suspended in New York City during the pandemic. The inclusion criteria were (1) formal diagnosis of PD from a neurologist and (2) the presence of action tremor in one or both hands, with a rating of >1 on the Movement Disorders Society–sponsored Revision of the Unified Parkinson’s Disease Rating Scale part III item 3.16 (rating of tremor). Exclusion criteria were: (1) history of comorbid neurological conditions, that is, including stroke or other neurodegenerative disease, (2) acute orthopedic conditions on the dominant hand, (3) chronic orthopedic conditions affecting the ability to write, (4) implantation of a pacemaker or deep brain stimulator, or (5) inability or unwillingness of the participant or legal guardian to give written informed consent. Individuals were recruited from the community via flyers and neurologists from Columbia University Irving Medical Center were informed of our study and could refer their patients.

### Ethical Considerations

This study was approved by the institutional review board at Teachers College, Columbia University (IRB#19-266) and all participants signed informed consent. Privacy and confidentiality standards were protected throughout the research study, and all study data collected were deidentified. Participants were compensated US $50 for their participation in this study.

### Emma Watch Device

The Emma Watch is a lightweight watch-like device worn around the dominant wrist. The device uses 5 small linear resonant actuators, each with a 205 Hz vibration frequency and controlled by a driver with an auto resonance engine. The vibration's strength and modulation are controlled via a Microsoft Surface tablet app (Microsoft), which connects to the Emma Watch via Bluetooth. The vibration is initiated at the start of movement and delivered throughout the task. The linear resonant actuators were run by a haptic driver at 100%, 64.7%, and 50% duty cycle, producing 3 vibration amplitudes: ~1.51 g (high intensity), ~0.38 g (low intensity), and 0 g (no vibration) modulated with a 500-millisecond/500-millisecond on/off vibration cycle in a counterbalanced order.

### Study Design

Individuals participated in two 90-minute sessions, performed on different days, but only 1 day for each participant was analyzed for this pilot study. Participants were tested within 1 hour after administration of their regular disease-specific medication. All participants donned the Emma Watch, along with 3 wearable inertial measurement units (APDM Opal sensors; APDM Wearable Technologies), one on each wrist and their trunk. All assessments were video recorded using a GoPro camera (GoPro). Participants completed baseline clinical assessments, along with a series of handwriting and fine motor assessments, with the Emma Watch counterbalanced on 3 vibration conditions. The ~0.38 g (low) is a very mild vibration and acted as a control condition [21].

### Assessments

We obtained demographic information from each participant, including age, gender, hand dominance, current list of medications, and education level. The effect of tremor on daily living activities was evaluated using the Bain and Finley Activities of Daily Living Scale [22] and self-reported hand function was evaluated using the Manual Ability Measure [23] (see Multimedia Appendix 1).

### Tablet Analysis

Participants completed 3 repetitions of handwriting and drawing tasks using a stylus and a digitizing tablet (Microsoft Surface). Participants were instructed to conduct the tasks as quickly and accurately as possible. Tasks included drawing horizontal or vertical lines, tracing a star, spiral, writing “elelelel” in cursive, and printing a standardized sentence. Each task was completed 3 times at 3 different vibration intensities: with the Emma Watch counterbalanced at a high intensity, a low intensity (sham), and their baseline with no vibration. Digital analysis captured kinematic [24], dynamic, and spatial attributes of drawing and writing samples to calculate mathematical indices that quantify upper limb motor function. The tablet recorded the pen’s x- and y-position and timestamp without wires or other attachments. The Windows app stored and converted data into readable files for analysis.

Analysis of the tablet recordings was performed with Python 3.7 (Python Software Foundation). The pen’s position in the x- and y-direction was converted to Euclidian distance at each point. The median sampling frequency of the x- and y-coordinate data was 142 Hz. To ensure a constant sampling frequency, the distance was resampled to a constant 142 Hz, using linear interpolation. Pointwise velocity was calculated as pointwise Euclidian distance divided by pointwise time interval. Velocity data were smoothed using a 3.5-Hz cut off, 5th order, low-pass Butterworth filter to remove high frequency fluctuations. We used this cut off to quantify slow movements related to writing. Measures calculated were (1) duration (seconds) of each drawing task, defined as the time taken to complete the drawing, from stylus down to stylus up, including pauses between strokes; (2) number of pauses, where a pause is any lift off of the stylus while drawing; (3) pause duration (seconds), defined as the sum of all pause times; (4) mean velocity (pixels/second) of each drawing task, defined as the mean of each pointwise velocity; and (5) fluency, defined as the number of local maxima (peaks) in the velocity profile. These peaks were found by comparison of neighboring values without any threshold.

### Accelerometry Analysis

Preprocessing of the APDM Opal Sensor data was carried out in MATLAB (MathWorks R2020A). Raw signals from the accelerometers were set to horizontal and vertical coordinates by the sensors’ preexisting algorithms, which use the magnetometers and gyroscopes to identify x-, y-, and z-reference positions. Right and left wrist streams were extracted and processed in MATLAB. Accelerometry streams, sampled at 128 Hz, were filtered through a 3.5 Hz cut off, zero-phase, low-pass Butterworth filter. This filtering profile is consistent with previous uses of APDM inertial measurement unit sensors in identifying anticipatory postural adjustments. The accelerometer and video data were synched through a series of claps, performed to identify the start of each drawing task. The start of a clap was designated if it met a power threshold of 0.17 in the accelerometry stream and was followed by 110 consecutive data points above the threshold.

The processing of the APDM Opal Sensor data was done using R (R Foundation for Statistical Computing) and Python (Python Software Foundation). Further, 32 metrics were calculated for each of the 3 axes of acceleration and jerk signals using *mhealthtools* [25] *R* package. These include the mean, complexity, mobility, roughness, rugosity, Shannon entropy of the frequency probability distribution, mean frequency, and the energy present in the twenty-four 0.5 Hz-bands between 2 Hz and 12 Hz. The signal’s fluency was computed in a similar way to the tablet drawings streams.

### Statistical Analysis

Statistical tests were conducted using R (version 4.02). Linear mixed-effects models were used to compare means of task outcomes (duration, number of pauses, pause duration, mean velocity, and number of peaks) between vibration intensities (zero, low, and high) within the 5 tasks (rectangle, spiral, star, elelelel, and handwriting; see Table 1). Linear mixed effects models used vibration frequency as a categorical variable, generated a random intercept, and used the “*lme4*” package on R [26].

**Table 1:** Duration, velocity, and peaks for tablet tasks.

Task				No vibration, mean (SD)		Low intensity, mean (SD)		High intensity, mean (SD)
**Spiral**								
	Duration (seconds)			38.8 (23.7)		37.2 (20.4)		37.1 (21.1)
	Pause number			1.6 (2.8)		0.8 (1.2)		1.2 (2.0)
	Pause time (seconds)			0.014 (0.02)		0.008 (0.01)		0.012 (0.02)
	Scaled peaks (peaks/s)			2.2 (0.1)		2.2 (0.2)		2.2 (0.2)
	Number of peaks			85.8 (50.6)		85.3 (52.1)		82.4 (48.1)
	Mean velocity (pixels/s)			168.0 (65.1)		171.0 (73.6)		171.8 (71.2)
**Handwriting**								
	Duration (seconds)			18.2 (4.9)		17.3 (5.0)		18.3 (5.7)
	Pause number			25.2 (1.2)		24.6 (1.9)		24.4 (2.0)
	Pause time (seconds)			0.2 (0.03)		0.2 (0.02)		0.2 (0.03)
	Scaled peaks (peaks/s)			1.4 (0.2)		1.5 (0.2)		1.5 (0.2)
	Number of peaks			25.2 (8.3)		24.9 (7.3)		26.5 (9.0)
	Mean velocity (pixels/s)			201.5 (80.7)		195.3 (78.3)		195.1 (68.9)
**APDM Spiral**								
	**Acceleration**							
		Complexity	23.5 (3.7)		22.2 (3.4)		23.3 (2.7)	
		Roughness	0.03 (0.05)		0.08 (0.1)		0.06 (0.1)	
		Rugosity	0.009 (0.006)		0.01 (0.008)		0.012 (0.008)	
		Mobility	15.6 (2.4)		14.4 (1.9)		15.0 (1.5)	
		Frequency	2.3 (0.5)		2.1 (0.4)		2.1 (0.3)	
		Entropy	0.57 (0.04)		0.55 (0.04)		0.56 (0.02)	
		Peaks	172.0 (102.6)		135.1 (82.2)		148.5 (82.7)	
		Normalized peaks	4.7 (0.5)		4.2 (0.5)		4.2 (0.5)	
	**Jerk**							
		Complexity	41.8 (10.1)		40.3 (21.1)		40.0 (10.3)	
		Roughness	60.5 (122.5)		121.4 (200.3)		92.9 (173.0)	
		Rugosity	0.21 (0.12)		0.32 (0.22)		0.30 (0.22)	
		Mobility	25.1 (4.3)		23.5 (4.6)		24.0 (2.7)	
		Frequency	3.5 (0.6)		3.3 (0.5)		3.4 (0.3)	
		Entropy	0.60 (0.03)		0.59 (0.04)		0.60 (0.03)	

## Results

In total, 9 people with PD were tested: 4 males and 5 females with a mean age of 67 (SD 9.4) years. The results showed no differences in any of the outcomes of interest measured with the tablet (duration, mean velocity, number of peaks, pause time, and number of pauses), or with APDM (acceleration and jerk). The only exception was APDM normalized acceleration peaks, where a main effect of intensity was found [sham: 4.67 (0.52), low: 4.18 (0.53), and high: 4.21 (0.50)]. However, a post hoc Tukey test revealed no pairwise-differences between frequencies.

In total, 3 out of 9 participants reported noticeable or marginal improvement, 4 out of 9 reported enjoyment in device use; 8 out of 9 reported device comfort, and no problems with the device were reported. For those individuals who reported perceived improvement, a stratified sample of tremor severity should be used in future studies to clarify which participants may garner efficacious results.

**Figure 1:**
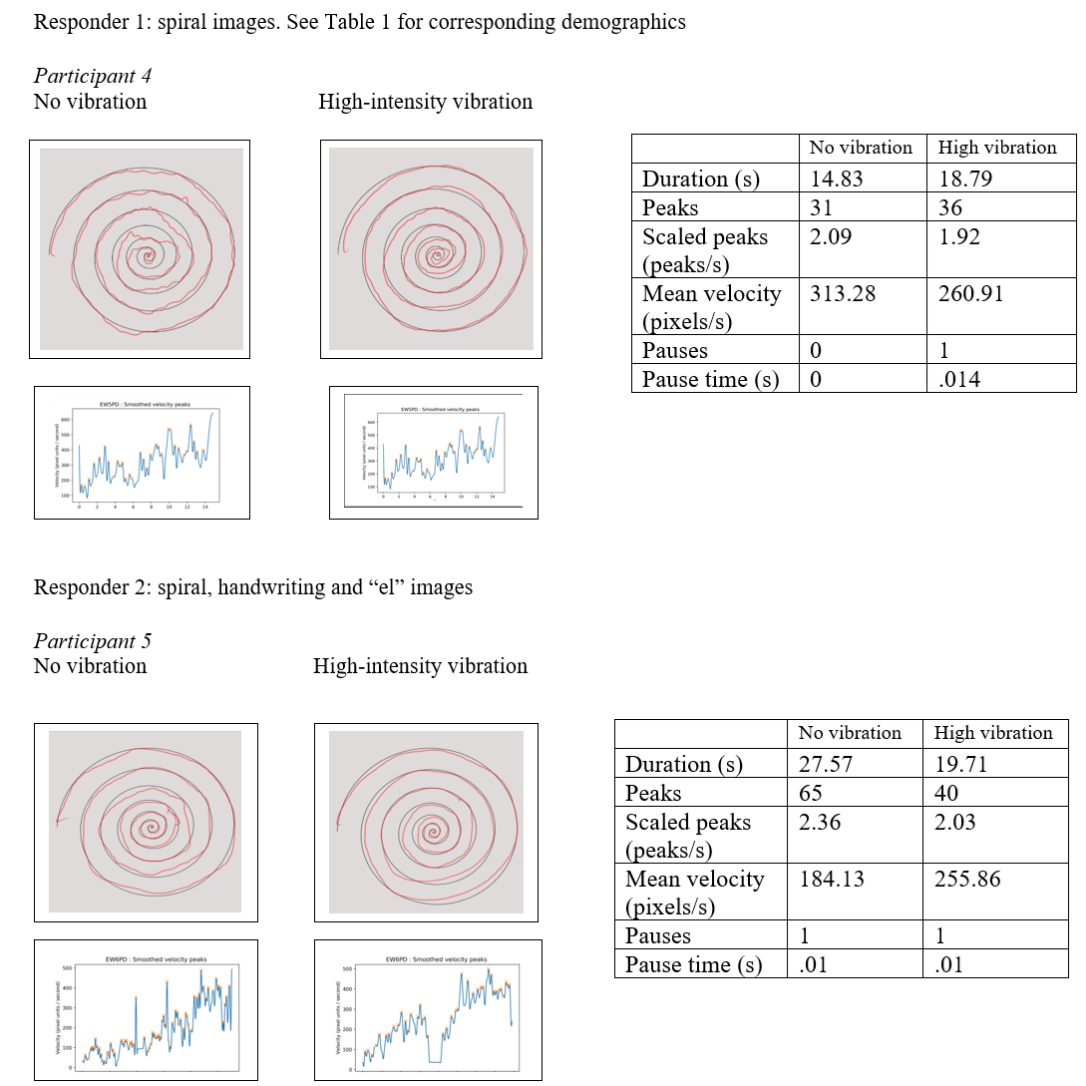
This figure represents responders to high vibration for spiral drawings.

Figure 1 shows 2 representative participants who demonstrated a visual improvement on the spiral tasks in the high versus sham condition, with corresponding objective data of task duration, pause duration, pause count, mean velocity, and number of peaks. Participant 4 reported perceived improvement and expressed greater functional difficulty on their assessment or baselines scores (see Multimedia Appendix 1). Alternatively, participant 5 had a visual improvement in spiral quality; however, did not report perceived improvement. This visual difference was observed within a small subset of participants, some of whom reported perceived improvement. Reports included a “benefit on straight lines,” “difference on spiral and star,” that “continuous motion (was) easier,” and the “most impact of tremor (was on) spiral.”

## Discussion

### Principal Findings

This pilot study investigated the use of the Emma Watch during fine motor and handwriting tasks for PD-induced action tremor. We recruited a range of people with PD with varying degrees of action tremor. The participants performed 5 different handwriting tasks on a digitized tablet with the use of the Emma Watch on the dominant wrist. The Emma Watch provided vibration at high, low, and zero intensity in a counterbalanced order. The device was found to be safe, and there were no adverse reactions. When interpreting our quantitative results, it is important to consider the small sample size of 9 people with PD. There was a main effect for differences in normalized acceleration peaks measured by APDM Opal sensors, however a post hoc Tukey test revealed no pairwise differences between vibration intensities. Visual differences were observed within a small subset of participants, some of whom reported perceived improvement.

### Comparison to Prior Work

The basal ganglia play a critical role in automatic and volitional motor performance, making automatic motor tasks, such as walking and handwriting difficult for people with PD. In addition, the loss of dopamine from PD causes a decrease in activation of the circuity that runs through the sensorimotor cortex and the basal ganglia; thus, increasing the response from the sensory system may alter the feedback loop and improve automatic motor responses [27]. In previous studies, the use of somatosensory cues has been successful in compensation strategies for improving gait impairments for people with PD [28]. Peripheral vibration is a form of sensory stimulation that provides proprioceptive input and may improve neuromotor function in people with sensorimotor deficits [18]. In a previous study in people with PD who had resting tremor, use of full body vibrotactile stimulation via 4 wearable devices on both wrists and ankles was found to be safe, feasible, and to possibly attenuate resting tremor [20]; however, people with action tremor were not included.

The Emma Watch has been hypothesized to mitigate tremor by mediating sensory signals in the cerebello-thalamo-cortical circuit, which has been linked to the origination of action tremor [29,30]. A recent study found that 80 Hz of vibration was sufficient to demonstrate improved motor performance as well as decreased beta oscillatory activity over the contralateral sensorimotor cortex compared to 20 Hz in people with PD, suggesting that higher frequency peripheral vibration increases the excitability of the sensorimotor cortex [15]. In another study, randomized frequency peripheral vibration from the TheraBracelet, when used in conjunction with a therapy, was found to be safe and feasible for upper extremity motor recovery post stroke [31]. In addition, in a previous study in people with PD, the Emma Watch, at 200 Hz at 60 bpm modulation, was found to have a significant improvement on movement speed and precision of motor performance during tracing motor control tasks when compared to 200 Hz at 20 bpm modulation [17]. Therefore, we hypothesized that the high intensity condition would be sufficient to improve motor function and performance in our study. As per recent evidence suggestions, sensory based strategies, including high-intensity vibration, may potentially improve motor learning and motor performance on automatic motor tasks [15,17,31]. This study is one of the first to report high-intensity vibration during fine motor and handwriting tasks for the primary aim of reducing action tremor in people with PD.

### Strengths and Limitations

While we found no difference in task performances with the device on versus off in our 9 participants, a visual difference in accuracy was observed within a small subset, who further reported perceived improvement in handwriting or drawing skills. It is unclear why some participants with action tremor experienced improvements while others did not. Perhaps it may be that individuals who have a lower perceived hand function ability, or greater tremor severity, perform better with use of the device.

There are multiple limitations in this study that should be considered when interpreting the results. First, the results of this study are limited due to the small sample size as we did not reach our planned recruitment goal due to the COVID-19 pandemic; therefore, future studies would benefit from a larger sample size to increase the statistical power. Second, other limitations included technical difficulties spanning app failure, device malfunction, and data-saving issues. Unfortunately, these technical difficulties further reduced the amount of data available.

Third, the Emma Watch was used for a relatively short time period, and hypothetically, it is possible that length of time under stimulus may affect its efficacy. Alternatively, a learning effect might develop while using the Emma Watch as the body adapts to both the vibration stimulus and to the repetition of drawing the same 5 tasks multiple times. Lastly, handwriting was performed on a tablet with a stylus, which has notable differences to handwriting performance using pen and paper, such as paper orientation and feedback from the pen. However, kinematic analysis could not be performed without use of a digitized tablet [24].

### Future Directions

Future studies should consider focusing on individuals with greater tremor severity, as 1 participant in our study who demonstrated visual improvement had an action tremor of 2 and was at Hoehn and Yahr Stage 3. Additionally, the vibration may need to be individually tailored to the participant to maximize benefits. A stratified sample of tremor severity should be used to clarify which participants may benefit from this or similar devices. Future studies may also consider including individuals with action tremor who have a diagnosis of Essential Tremor, as there may be a difference in the response generated from the Emma Watch. According to Chen et al [32], there was a difference in the velocity of spiral drawing between patients with essential tremor and those with PD who had similar severity in their action tremor. We initially did include people with essential tremor in our study, and wanted to compare the 2 populations; however, due to technical difficulties and the COVID-19 pandemic halting human research, we did not have sufficient data to analyze and compare to people with PD.

While we recognize that the small sample size and technical difficulties limit the interpretation of our results, there were visually depicted and subjectively reported improvements for a small subset of participants that are important to recognize. As action tremor severely affects quality of life and functional independence for people with PD, it is increasingly important to report on any interventions that may potentially improve functional abilities [8]. Future studies must focus on finding safe and efficacious ways to address this clinical need and should explore the efficacy of combining the Emma Watch with task-specific training or other intervention tools, as this may attenuate action tremor. However, the Emma Watch efficacy in improving fine motor or handwriting skills as a stand-alone tool remains to be demonstrated.

## Appendix

Multimedia Appendix 1 Further information on participant demographics, means of tasks outcomes between intensities, responders for “elelelel” drawings, the equations used in the APDM analysis, and participant quotes.

## Abbreviations

PD: Parkinson disease

## Data Availability

The APDM data and tablet data sets generated during and/or analyzed in this study are available from the corresponding author on reasonable request.

## References

[1] Jankovic J (2008). Parkinson's disease: clinical features and diagnosis. J Neurol Neurosurg Psychiatry.

[2] Simon DK, Tanner CM, Brundin P (2020). Parkinson disease epidemiology, pathology, genetics, and pathophysiology. Clin Geriatr Med.

[3] Armstrong MJ, Okun MS (2020). Diagnosis and treatment of Parkinson disease: a review. JAMA.

[4] Abusrair AH, Elsekaily W, Bohlega S (2022). Tremor in Parkinson's disease: from pathophysiology to advanced therapies. Tremor Other Hyperkinet Mov (N Y).

[5] Helmich RC, Toni I, Deuschl G, Bloem BR (2013). The pathophysiology of essential tremor and Parkinson's tremor. Curr Neurol Neurosci Rep.

[6] Lenka A, Jankovic J (2021). Tremor syndromes: an updated review. Front Neurol.

[7] Louis ED, Levy G, Côte LJ, Mejia H, Fahn S, Marder K (2001). Clinical correlates of action tremor in Parkinson disease. Arch Neurol.

[8] Heusinkveld LE, Hacker ML, Turchan M, Davis TL, Charles D (2018). Impact of tremor on patients with early stage Parkinson's disease. Front Neurol.

[9] Helmich RC, Dirkx MF (2017). Pathophysiology and management of Parkinsonian tremor. Semin Neurol.

[10] Muthuraman M, Heute U, Arning K, Anwar AR, Elble R, Deuschl G, Raethjen J (2012). Oscillating central motor networks in pathological tremors and voluntary movements. What makes the difference?. Neuroimage.

[11] Muthuraman M, Raethjen J, Koirala N, Anwar AR, Mideksa KG, Elble R, Groppa S, Deuschl G (2018). Cerebello-cortical network fingerprints differ between essential, Parkinson's and mimicked tremors. Brain.

[12] Wong JK, Viswanathan VT, Nozile-Firth KS, Eisinger RS, Leone EL, Desai AM, Foote KD, Ramirez-Zamora A, Okun MS, Shukla AW (2020). STN versus GPi deep brain stimulation for action and rest tremor in Parkinson's disease. Front Hum Neurosci.

[13] Gigante AF, Bruno G, Iliceto G, Guido M, Liuzzi D, Mancino PV, De Caro MF, Livrea P, Defazio G (2015). Action tremor in Parkinson's disease: frequency and relationship to motor and non-motor signs. Eur J Neurol.

[14] Dincher A, Schwarz M, Wydra G (2019). Analysis of the effects of whole-body vibration in Parkinson disease—systematic review and meta-analysis. PM R.

[15] Macerollo A, Palmer C, Foltynie T, Korlipara P, Limousin P, Edwards M, Kilner JM (2018). High-frequency peripheral vibration decreases completion time on a number of motor tasks. Eur J Neurosci.

[16] King LK, Almeida QJ, Ahonen H (2009). Short-term effects of vibration therapy on motor impairments in Parkinson's disease. NeuroRehabilitation.

[17] Macerollo A, Holz C, Cletheror D, Vega J, Moody J, Saul G, Paleyes N, Villar N, Korlipara P, Foltynie T, Limousin P, Zhang H, Kilner J (2020). Non-invasive intervention for motor signs of Parkinson's disease: the effect of vibratory stimuli. J Neurol Neurosurg Psychiatry.

[18] Lau RWK, Teo T, Yu F, Chung RCK, Pang MYC (2011). Effects of whole-body vibration on sensorimotor performance in people with Parkinson disease: a systematic review. Phys Ther.

[19] Buki E, Katz R, Zacksenhouse M, Schlesinger I (2018). Vib-bracelet: a passive absorber for attenuating forearm tremor. Med Biol Eng Comput.

[20] Tabacof L, Braren S, Patterson T, Fry A, Putrino D (2021). Safety and tolerability of a wearable, vibrotactile stimulation device for Parkinson's disease. Front Hum Neurosci.

[21] Jöbges EM, Elek J, Rollnik JD, Dengler R, Wolf W (2002). Vibratory proprioceptive stimulation affects Parkinsonian tremor. Parkinsonism Relat Disord.

[22] Bain PG, Findley LJ, Atchison P, Behari M, Vidailhet M, Gresty M, Rothwell JC, Thompson PD, Marsden CD (1993). Assessing tremor severity. J Neurol Neurosurg Psychiatry.

[23] Chen CC, Bode RK (2010). Psychometric validation of the Manual Ability Measure-36 (MAM-36) in patients with neurologic and musculoskeletal disorders. Arch Phys Med Rehabil.

[24] Thomas M, Lenka A, Pal PK (2017). Handwriting analysis in Parkinson's disease: current status and future directions. Mov Disord Clin Pract.

[25] Snyder P, Tummalacherla M, Perumal T, Omberg L (2020). mhealthtools: a modular R package for extracting features from mobile and wearable sensor data. J Open Source Softw.

[26] Bates D, Mächler M, Bolker B, Walker S (2015). Fitting linear mixed-effects models using lme4. J Stat Soft.

[27] Petzinger GM, Fisher BE, McEwen S, Beeler JA, Walsh JP, Jakowec MW (2013). Exercise-enhanced neuroplasticity targeting motor and cognitive circuitry in Parkinson's disease. Lancet Neurol.

[28] Nonnekes J, Ružicka E, Nieuwboer A, Hallett M, Fasano A, Bloem BR (2019). Compensation strategies for gait impairments in Parkinson disease: a review. JAMA Neurol.

[29] Helmich RC, Hallett M, Deuschl G, Toni I, Bloem BR (2012). Cerebral causes and consequences of Parkinsonian resting tremor: a tale of two circuits?. Brain.

[30] Lora-Millán JS, López-Blanco R, Gallego JÁ, Méndez-Guerrero A, de la Aleja JG, Rocon E (2019). Mechanical vibration does not systematically reduce the tremor in essential tremor patients. Sci Rep.

[31] Seo NJ, Woodbury ML, Bonilha L, Ramakrishnan V, Kautz SA, Downey RJ, Dellenbach BHS, Lauer AW, Roark CM, Landers LE, Phillips SK, Vatinno AA (2019). TheraBracelet stimulation during task-practice therapy to improve upper extremity function after stroke: a pilot randomized controlled study. Phys Ther.

[32] Chen KH, Lin PC, Yang BS, Chen YJ (2018). The difference in visuomotor feedback velocity control during spiral drawing between Parkinson's disease and essential tremor. Neurol Sci.

