# Supplementary material for: A Wearable Vibratory Device (The Emma Watch) to Address Action Tremor in Parkinson Disease: Pilot Feasibility Study
Source: JMIR Biomed Eng. 2023 Oct 23;8:e40433. doi: 10.2196/40433 (PMC11041244; doi:10.2196/40433)
Supplement: Multimedia Appendix 1 [file biomedeng_v8i1e40433_app1.docx]

**Multimedia Appendix 1**

This appendix contains further information on participant demographics, means of tasks outcomes between intensities, responders for “elelelel” drawings, the equations used in the APDM analysis and participant quotes.

**Table S1.** Demographics and clinical characteristics.

| **Participant** | **Hand Dominance** | **Most Affected Hand** | **Education** | **Sex** | **Age** | **H&Y** | **Action Tremor** | **Rest Tremor** | **Total MAM** | **Total ADL** |
| --- | --- | --- | --- | --- | --- | --- | --- | --- | --- | --- |
| 1 | Right | Right | Post Doctorate | Male | 67 | 2 | 2 | 2 | 129 | 41 |
| 2 | Right | Right | Bachelors | Female | 71 | 2 | 1 | 2 | 124 | 39 |
| 3 | Right | Right | Masters | Female | 73 | 1.5 | 2 | 2 | 122 | 37 |
| 4 | Right | Right | Some Graduate School | Male | 77 | 3 | 2 | 1 | 105 | 53 |
| 5 | Right | Left | Bachelors | Female | 81 | 2 | 1 | 1 | 107 | 42 |
| 6 | Left | Left | Masters | Female | 55 | 2 | 1 | 1 | 101 | 45 |
| 7 | Right | Both | Doctorate | Male | 69 | 3 | 1 | 1 | 119 | 41 |
| 8 | Right | Right | Post Doctorate | Female | 60 | 2 | 2 | 2 | 116 | 40 |
| 9 | Right | Left | Associates | Male | 54 | 2 | 1 | 1 | 119 | 34 |

ADL: Lower scores on this questionnaire suggest less difficulty with a given task (range 25-100)

(1) able to do the activity without difficulty, (2) little effort, (3) a lot of effort, (4) cannot do the activity by yourself

MAM: Higher scores suggest less difficulty with a given task (range 0-144)

(4 = easy, 3 = A little hard 2 = very hard, 1 = cannot do, 0 = almost never do)

Abbreviations: MAM- Manual Ability Measure; ADL-Bain and Findley Activity of Daily Living Scale Score (Percent)

**Table S2**. Means (SD) of duration, velocity, and peaks for tablet tasks.

| **Task** | **No Vibration** | **Low Vibration** | **High Vibration** |
| --- | --- | --- | --- |
| **Rectangle** |  |  |  |
| Duration | 5.7(3.7) | 5.4(2.9) | 5.5(3.4) |
| Pause Number | 1.7(4.2) | 1.4(3.3) | 1.3(3.4) |
| Pause Time | 0.014(0.03) | 0.017(0.03) | 0.012(0.03) |
| Scaled Peaks (peaks/s) (peaks/s) (peaks/s) | 1.3(0.3) | 1.4(0.4) | 1.3(0.6) |
| Number of Peaks | 7.4(5.0) | 7.4(4.3) | 6.7(4.8) |
| Mean Velocity | 364.2(97.7) | 396.1(222.9) | 396.6(211.5) |
| **Star** |  |  |  |
| Duration | 16.1(6.6) | 16.8(7.0) | 16.1(4.1) |
| Pause Number | 0.5(1.0) | 0.7(0.9) | 0.4(0.7) |
| Pause Time | 0.005(0.01) | 0.007(0.008) | 0.005(0.008) |
| Scaled Peaks | 1.8(0.2) | 1.7(0.3) | 1.8(0.3) |
| Number of Peaks | 29.9(17.0) | 29.3(16.6) | 29.4(10.1) |
| Mean Velocity | 166.7(47.7) | 163.2(44.3) | 158.0(36.2) |
| **Elelelel** |  |  |  |
| Duration | 5.6(2.1) | 5.7(1.7) | 5.4(2.7) |
| Pause Number | 0.1(0.1) | - | 0.1(0.2) |
| Pause Time | 0.0008(0.002) | 0 | 0.0014(0.003) |
| Scaled Peaks | 2.5(0.3) | 2.5(0.4) | 2.8(1.1) |
| Number of Peaks | 13.4(3.4) | 13.7(2.8) | 12.8(4.3) |
| Mean Velocity | 296.0(112.7) | 299.6(122.9) | 330.9(116.6) |

Duration (s) Pause Time (s) Scaled Peaks (peaks/s) Mean Velocity (pixels/s)

*Significant main effect p < .05

**Figure S1.** Represents “el” images to responders of the Emma Watch.

See Table S1 for Corresponding demographics

Responder 1: “el” images.

*Participant 4*

No vibration High-intensity vibration

|  | No Vibration | High Vibration |
| --- | --- | --- |
| Duration (s) | 2.41 | 2.87 |
| Peaks | 7 | 8 |
| Scaled Peaks (peaks/s) | 2.89 | 2.91 |
| Mean Velocity (pixels/s) | 657.80 | 619.51 |
| Pauses | 0 | 0 |
| Pause Time(s) | 0 | 0 |


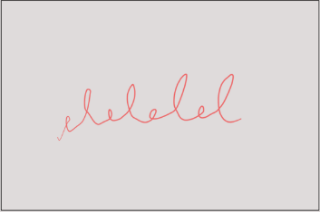

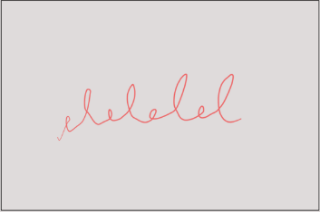


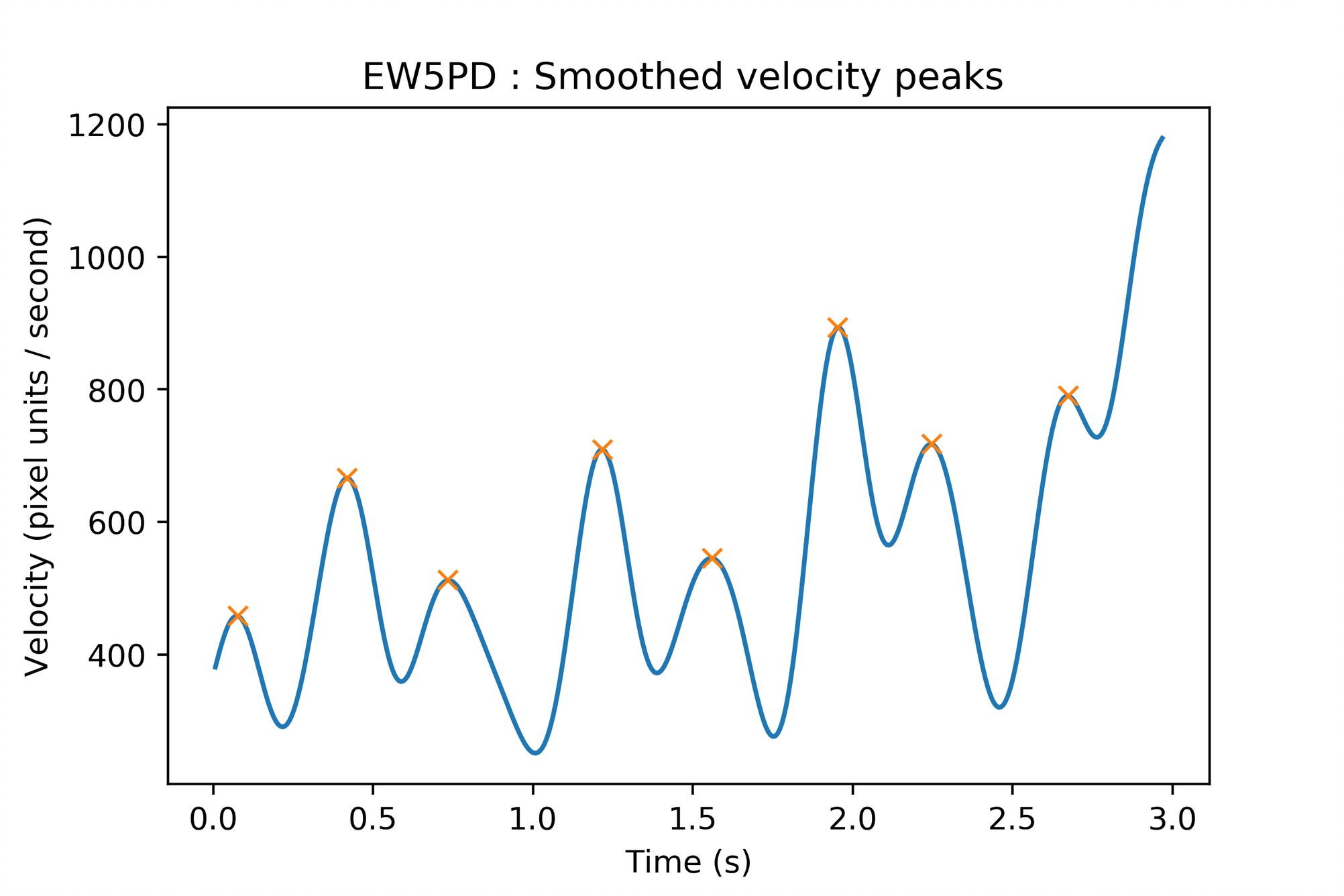


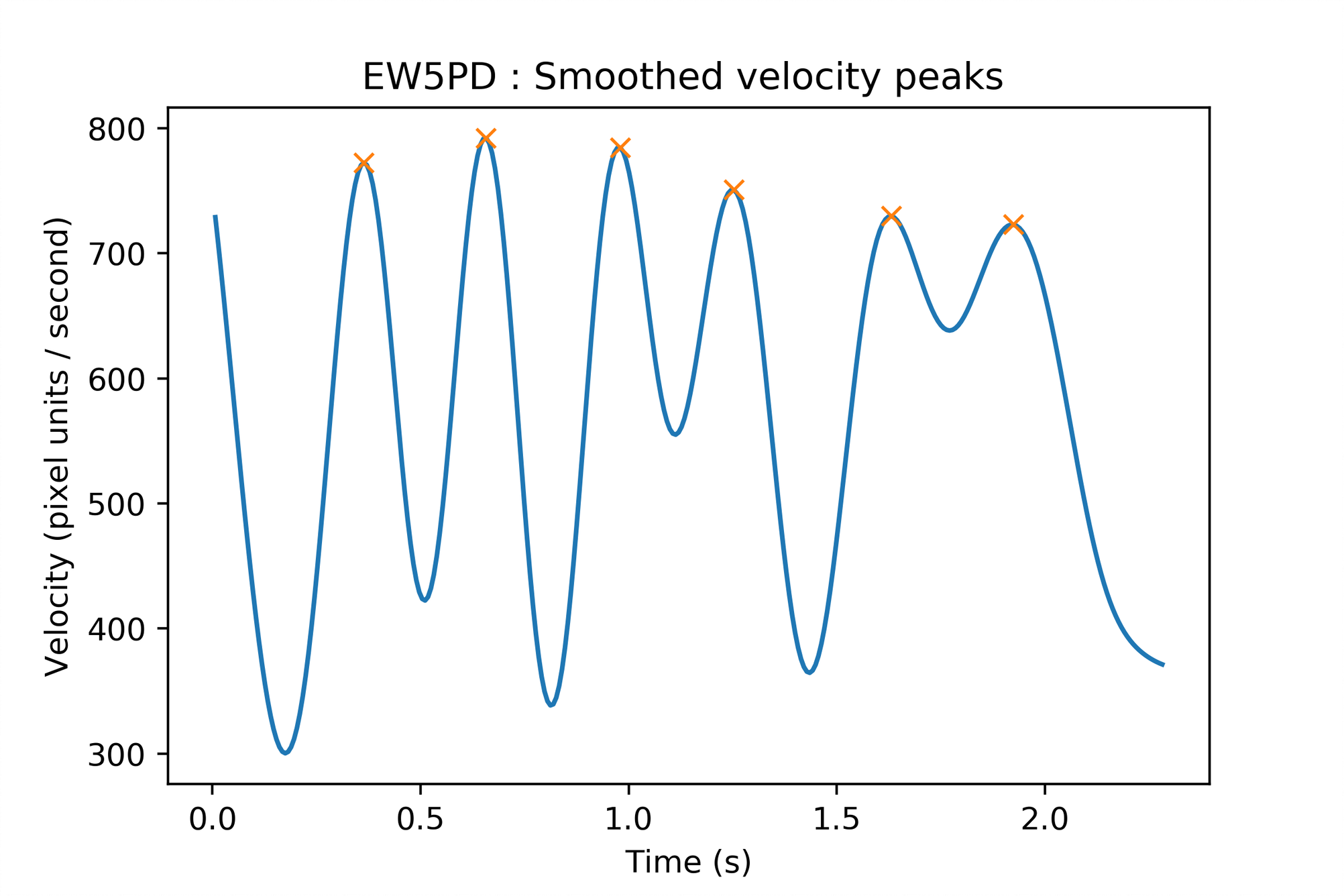


*Responder 2: “el” images*

*Participant 5*

No vibration High-intensity vibration

|  | No Vibration | High Vibration |
| --- | --- | --- |
| Duration (s) | 14.78 | 6.51 |
| Peaks | 92 | 67 |
| Scaled Peaks (peaks/s) | 1.76 | 2.30 |
| Mean Velocity (pixels/s) | 143.12 | 241.00 |
| Pauses | 1 | 0 |
| Pause Time (s) | .01 | 0 |


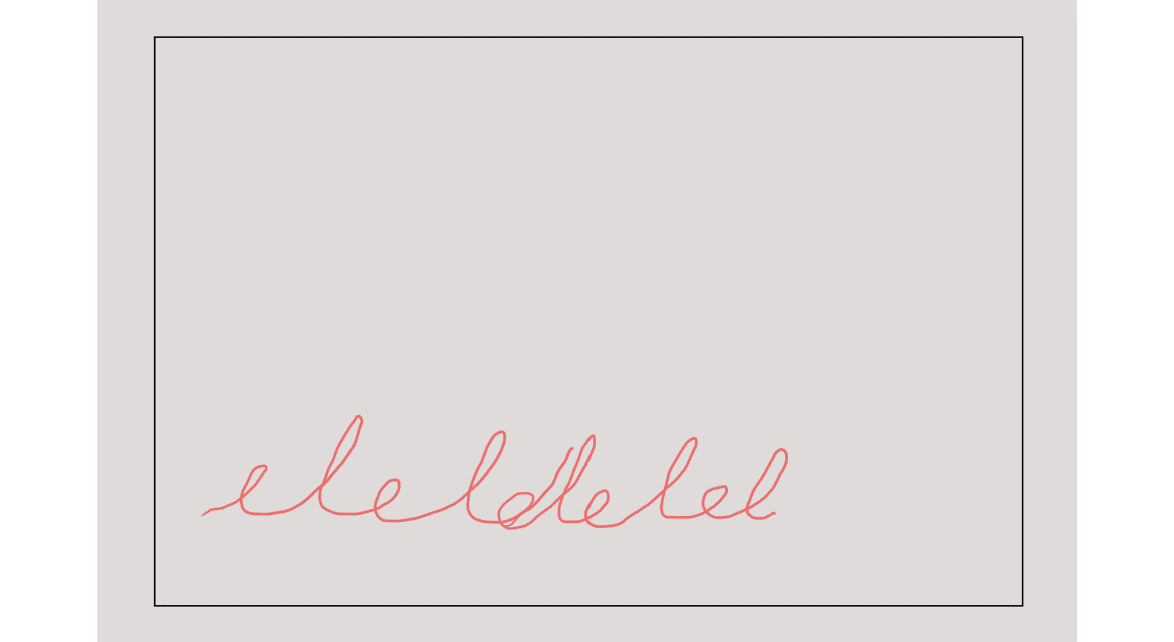


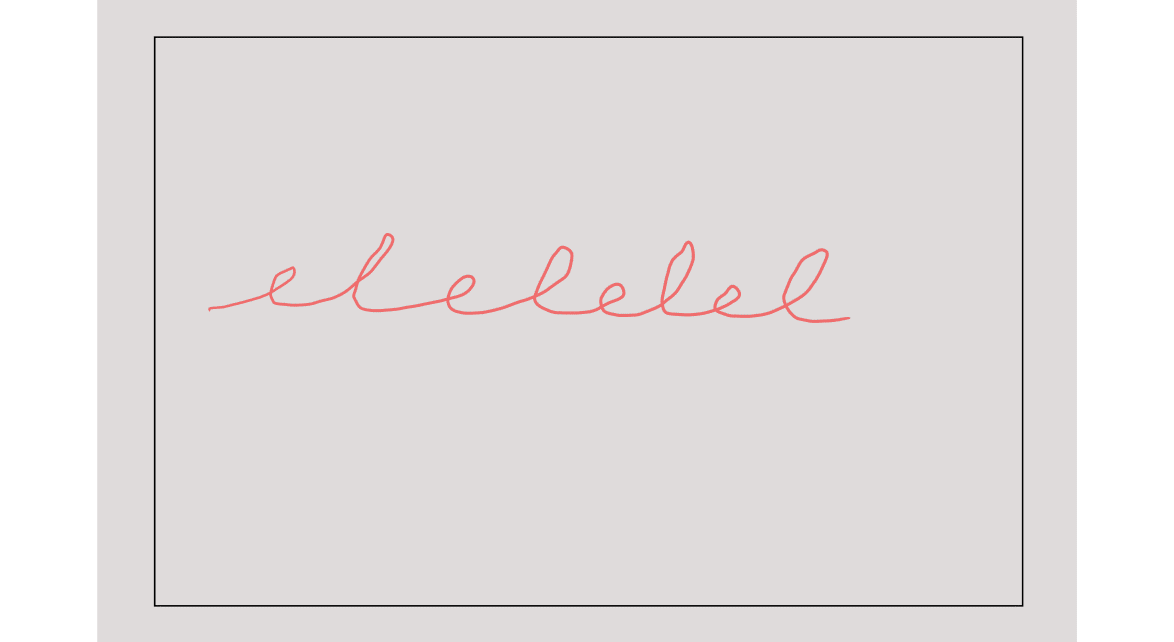


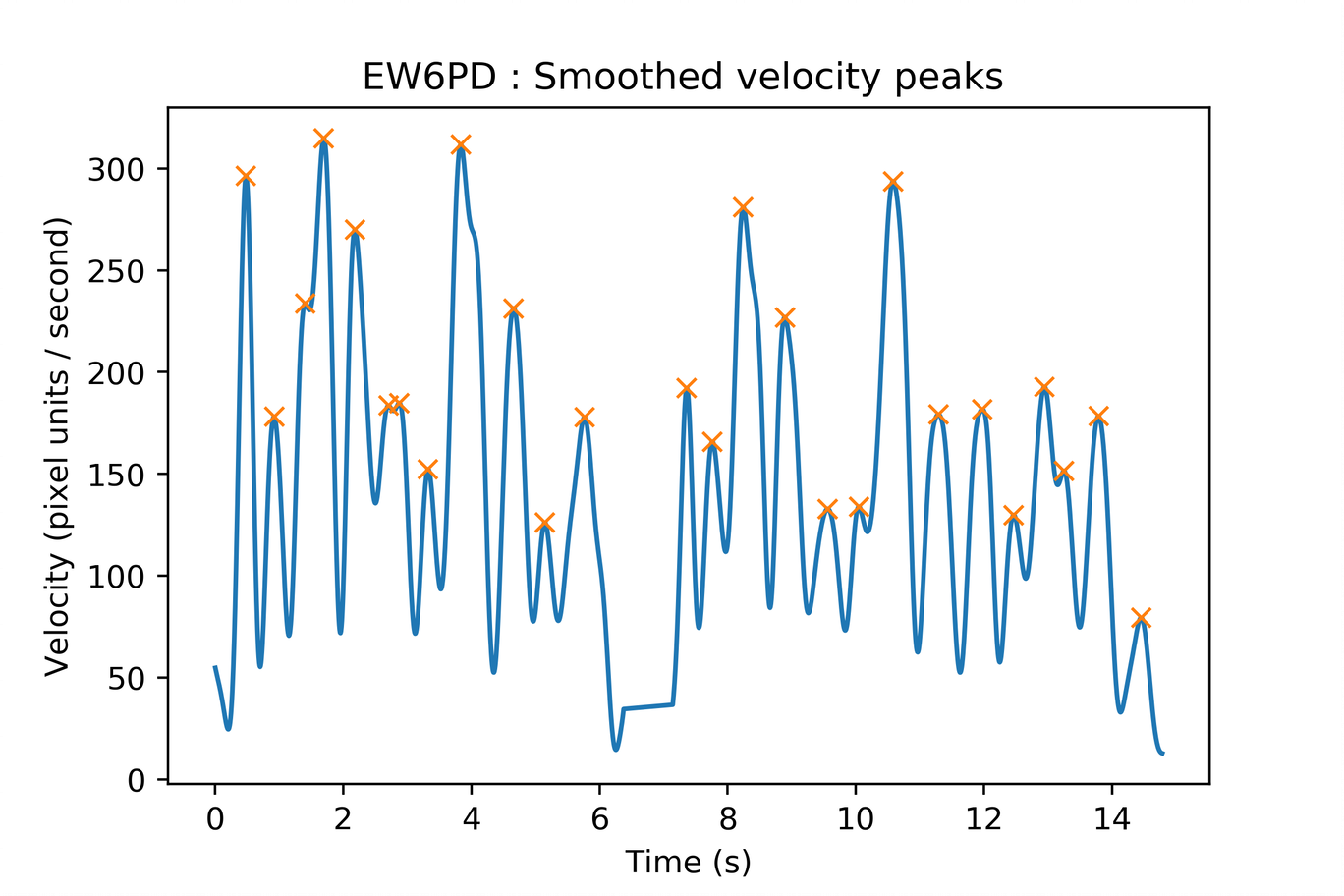


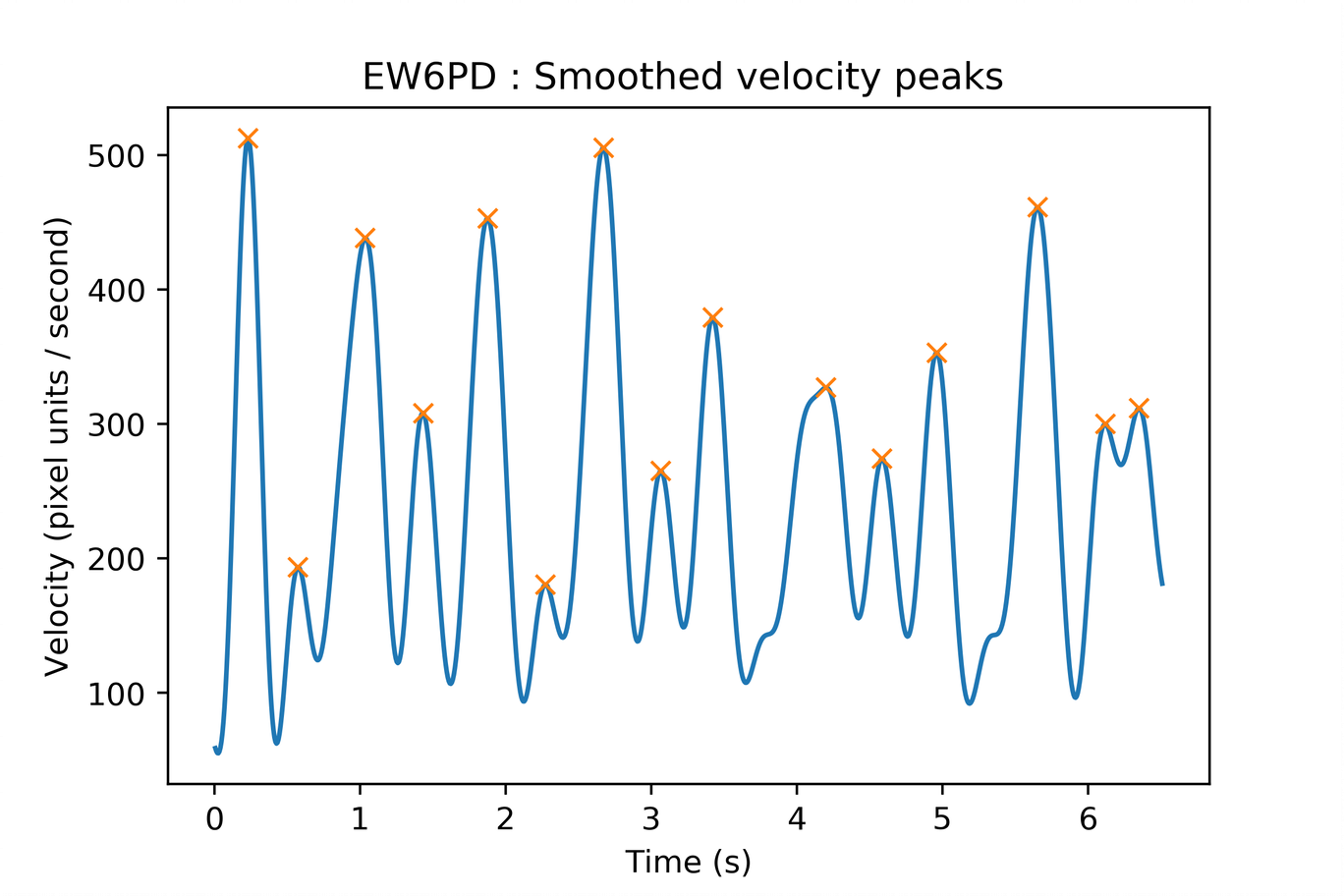


**Equations**. The following are the equations utilized during the APDM analysis

Equation S1:

$$\sqrt{\frac{Var(f_{s}^{2}\times\frac{d^{2}x}{dt^{2}})}{Var(f_{s}\times\frac{dx}{dt})}}$$

Equation S2:

$$\sqrt{\frac{Var(f_{s}\times\frac{dx}{dt})}{Var(x)}}$$

Equation S3:

$$\sum(freq\times\frac{pdf}{\sum pdf})$$

**Quotes.** The following are a variety of quotes that participants had after using the Emma Watch

| **Participant** | **Quote** |
| --- | --- |
| **1** | “(There) was a benefit on straight lines…(My) continuous motion (was) easier…most impact of tremor on (the) spiral…. coordination (was) easier…(I) Would happily wear (the) device to reduce tremor. |
| **4** | “Helpful printing sentence” |
| **6** | "I think it (Emma Watch) distracts my brain; the vibration overrides the focus on what I want to do (but) can't do…It (was) a really cool experience, it gives me hope...I felt like it had a lasting effect." |
| **7** | “ (The Emma Watch) would be great if it could help with tremor. Concept very appealing. After using the watch while drinking from a coke bottle, there was less tremor and support was not needed.” |
